# Update on the Treatment of Non-Small Cell Lung Carcinoma (NSCLC)

**DOI:** 10.3390/jcm14196960

**Published:** 2025-10-01

**Authors:** Yousif A. Kariri

**Affiliations:** Department of Clinical Laboratory Sciences, Collage of Applied Medical Sciences, Shaqra University, Shaqra 11961, Saudi Arabia; ykuriri@su.edu.sa

**Keywords:** non-small lung carcinoma, adverse effects, efficacy, tyrosine kinase inhibitors, epithelial growth factor receptor, clinical trials, phase IV

## Abstract

Non-small cell lung carcinoma (NSCLC) is a prominent type, with an 85–90% incidence in all lung cancer cases. The evidence for a particular therapy strategy for people with NSCLC is still inadequate. This review evaluates NSCLC therapies that have passed phase IV trials, emphasizing their efficiency and adverse effects. Crucial therapeutic approaches, including dacomitinib, lorlatinib, durvalumab, osimertinib, and rivaroxban, are discussed, highlighting their mechanisms of action, uses, and adverse effects. Immune checkpoint medications are recommended because of their specific activity and minimal adverse reactions. The review also investigates cooperation therapies, such as targeting immune checkpoint inhibitors and hemostasis, alongside chemotherapy, as they offer potential for future therapies. However, further research is needed to improve the safety and efficacy of current treatments, and to explore novel ways to achieve better long-term outcomes.

## 1. Introduction

Lung cancer is one of the most common leading cancers, resulting in high mortality and morbidity around the globe [1]. In 2022, lung cancer accounted for 1.57 million new cases in males, making it the most frequent cancer diagnosed globally, according to GLOBOCAN, lung cancer was the first most diagnosed cancer in males and the second most in females, with approximately 910,000 new cases [2]. Recently, lung cancer was demonstrated to have only a 25% probability of 5-year survival, one of the lowest survival rates of all cancers in 2024 [3]. Despite recent developments in lung cancer screening, diagnosis, prevention, and therapy, the significant connection between incidence and mortality indicates that the disease is still generally fatal.

The occurrence of lung cancer cases by gender and population is anticipated to be mainly determined by cigarette smoking. Although lung cancer has been generally more common among males who smoked more frequently in the past few decades, recent studies reveal an increasing incidence of lung cancer cases in women, especially among younger age groups, and a drop in lung cancer instances in men [4]. A study conducted in the United States reported that the incidence of lung cancer was higher in young women compared to young men [5], and it certainly indicates that this development does not just hinge on changing patterns in cigarette smoking [4]. Exposure to asbestos, as the main risk factor for the development of malignant mesothelioma, secondhand smoking, mineral and metal dusts, and radon are other recognized risk factors for lung cancer [6]. The United States’ lung cancer mortality rate has recently dropped by 59% for men, from its peak in men in 1990, and 36% for women, from its peak in women in 2002, due to the implementation of screening guidelines and an overall decrease in tobacco usage [3].

Based on cancer cells’ histology, lung cancer can be divided into two distinct groups: NSCLC and small cell lung carcinoma (SCLC). NSCLC is the dominant lung cancer type with 85–90% incidence in all lung cancer cases. Remarkably, NSCLC is a very severe disease characterized by high rates of metastasis and recurrence, and composed of several histological subtypes, including large-cell carcinoma, lung squamous carcinoma, and lung adenocarcinoma [7]. Another histological subclassification classified NSCLC according to cell types into squamous NSCLC (constituting up to 30% of NSCLC cases), and non-squamous carcinoma NSCLC, which may be further classified into adenocarcinoma (constituting up to around 40% of NSCLCs), big cell carcinoma, and other cell types [8].

The stage of NSCLC at diagnosis impacts significantly on treatment options and prognoses. To illustrate, in the early stages, surgery is a highly effective therapeutic option, whereas it is not recommended once metastasis occurs. Moreover, NSCLC treatment is often multimodal, including surgery, radiotherapy, and chemotherapy; for stage 4, systemic treatment is the current standard of care, but radiotherapy and surgery may also have a role (i.e., palliative treatment) [9,10]. Despite the utilization of conventional chemotherapeutic drugs for advanced lung carcinoma cases, some limitations reduce their effectiveness in cancer treatment, such as the development of drug resistance, non-specific targeting, and low bioavailability [11]. Hence, the most effective advance in recent years in NSCLC therapy is the focus on molecular targeted therapy. Exploring the genomic drivers is the key to identifying the etiology and understanding the pathogenesis of NSCLC. Tyrosine kinase inhibitors (TKIs) are a new group of treatments that have emerged as a result of the identification of activating mutations of epidermal growth factor receptor (EGFR) in patients with lung adenocarcinoma [12]. These therapeutic strategies open up new horizons in terms of patients’ clinical management, as targeting EGFR mutations can approximately double the survival rate compared to conventional chemotherapy [13].

## 2. Pharmacological Approaches to NSCLC Therapy

### 2.1. Molecular Pharmacological Treatments

**Figure 1:**
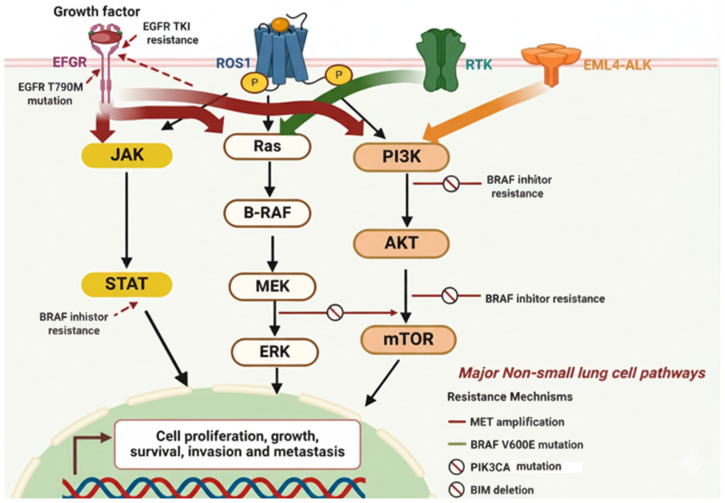
**Summary of the major TKIs mutation pathways involved in NSCLC**. JAK-STAT, RAS, and PI3K-AKT are three key signaling pathways in NSCLC. These pathways are well-known regulators of cell cycle development, growth, and apoptosis/cell survival; dysregulation is a common feature of human cancers. Modifications in essential pathways will influence DNA methylation modifications, such as elevated DNA methyltransferases (DNMTs), DNA post-translation, and reduced ten-eleven translocation methylcytosine dioxygenases (TETs), enabling amplification of mesenchymal homology box 2 (MEOX2), which has a negative association with patient survival. As a consequence, the expression of key cell cycle checkpoints and tumor suppressor biomarkers (P53, P21, and other related biomarkers) will decrease to allow cancer cell proliferation and survival. Tyrosine kinase inhibitors (TKIs) are a class of drugs specifically designed to target major genomic mutations that act as oncogenic drivers in non-small cell lung cancer (NSCLC). These mutations lead to the overactivation of key signaling pathways, which in turn drive uncontrolled cell proliferation, growth, and survival. As shown in the figure, the primary genomic mutations targeted by TKIs include the following: EGFR mutations: These are common mutations in the epidermal growth factor receptor (EGFR) gene. TKIs inhibit the signaling of the mutated EGFR protein, thereby blocking the downstream signaling pathways like RAS-RAF-MEK-ERK and PI3K-AKT-mTOR. The figure also shows the EGFR T790M mutation, a common resistance mechanism that requires a different type of TKI. ROS1 fusions: A genetic rearrangement involving the ROS1 gene that leads to a constitutively active kinase, which is targeted by specific TKIs. EML4-ALK fusions: A gene fusion that creates an oncogenic protein with constant kinase activity, which is a major target for ALK inhibitors, a type of TKI. The figure also highlights other genomic changes that can act as either primary drivers or resistance mechanisms, such as BRAF V600E mutation, MET amplification, and PIK3CA mutations, all of which can be targeted by specific inhibitors to disrupt the cancer cell’s signaling.

The increasing prevalence of TKI resistance has challenged the initial expectations and paved the way for the gradual development of novel medications that target multiple molecular modifications and pathways. Recently, patients diagnosed with advanced metastasis stages of NSCLC have shown mutations in several genes, including EGFR, c-ros oncogene 1 (ROS1), anaplastic lymphoma kinase (ALK), human epidermal growth factor receptor (HER2), and Kirsten rat sarcoma virus (KRAS), suggesting that mutations in these genes can be used as therapeutic targets [14,15,16]. Interestingly, finding a targetable mutation might assist patients in better understanding their prognosis and the progression of their medical condition, in addition to influencing clinical decision-making and therapy selection. However, to date, only medications that target EGFR or ALK mutations, as well as ROS and BRAF mutations, have been approved for clinical trials and treatments. To specify, patients with ALK modifications are recommended to be treated with brigatinib [17], or alectinib [18], whereas those with ROS1 modifications are treated with crizotinib [19]. Likewise, patients with lung adenocarcinoma harboring EFGR mutation are generally treated with osimertinib [20]. The pathways for these mutations are summarized in Figure 1. The recommendation of using these molecules, which target specific mutations, will increase in the future, driven by extensive ongoing research that aims to develop NSCLC target therapies. Furthermore, research on inhibitors with other targets is also ongoing [21]. Despite the initial effectiveness of the molecular targeted therapy approach, tumor heterogeneity and epigenetic alterations frequently lead to acquired drug resistance, which significantly reduces therapeutic efficiency in NSCLC [22,23]. Therefore, developing novel molecular targets for NSCLC is essential to improve patient outcomes.

### 2.2. Immune Target Pharmacological Treatments

Immunotherapy approaches that focus extensively on immune checkpoint inhibitors are another therapeutic option for NSCLC that is very successful and significantly safer for patients [24,25]. Immune checkpoint inhibitor-based treatments targeting PD-1 and PD-L1 are common therapies for advanced or metastatic NSCLC that lacks identifiable genetic causes [26]. Moreover, prior studies have indicated that combining immunotherapy with other medications or therapeutic modalities may be beneficial [27,28,29].

Although there have been significant improvements in NSCLC treatment through several options to explore the best therapeutic applications, the treatment of NSCLC is still controversial and needs improvement. This review summarizes the pharmacological effects of agents that completed phase IV clinical trials in the last 5 years for the treatment of NSCLC.

A review of recent studies on anti-PD-1 and anti-PD-L1 immune checkpoint inhibitors for non-small cell lung cancer (NSCLC) reveals nuanced differences in their efficacy and safety profiles [30]. While both classes of drugs have demonstrated a significant overall survival advantage compared to chemotherapy in second- and later-line treatments, subtle distinctions become apparent upon closer examination. A meta-analysis focusing on efficacy and safety in relapsed or refractory advanced NSCLC found that anti-PD-1 agents ranked highest for overall survival, with anti-PD-L1 agents following [31]. However, another systematic review specifically examining the safety of these inhibitors found that PD-L1 inhibitors might be a safer option regarding grade ≥3 treatment-related adverse events, particularly pneumonia and pneumonitis, when used alone or in combination with chemotherapy. A separate study further explored these differences, suggesting that anti-PD-1 agents may show a higher response rate and a better outcome when combined with chemotherapy for first-line treatment, especially for patients with specific PD-L1 expression levels. Conversely, the same study noted that anti-PD-L1 agents were associated with less severe immune-related adverse events [32]. These findings suggest that while both drug classes are effective, the choice between them may depend on a balance between desired efficacy and a patient’s tolerance for potential side effects.

## 3. Completed Phase IV Clinical Trials for NSCLC Treatment

### 3.1. Dacomitinib

Dacomitinib, developed by Pfizer, is a multi-kinase receptor regulator used for NSCLC therapy with EGFR activating mutations. Dacomitinib was approved as a therapeutic agent for metastatic NSCLC by the United States Food and Drug Administration in 2018 [33].

Dacomitinib is a permanent small-molecule inhibitor that efficiently regulates the EGFR family’s tyrosine kinase activity by targeting EGFR gene mutation, especially exon 19 deletions and exon 21 L858R point mutations [34,35]. Dacomitinib achieves permanent inhibition by forming covalent bonds with cysteine residues located inside the catalytic domains of HER receptors [36]. However, dacomitinib’s metabolic pathway is controlled by cytochrome p450 (CYP) CYP2D6 and CYP2A4, differing from other EGFR-TKIs, which are controlled by CYP3A4. This diversity in metabolic pathways might result in variable interactions among medications and pharmacological profiles.

**Figure 2:**
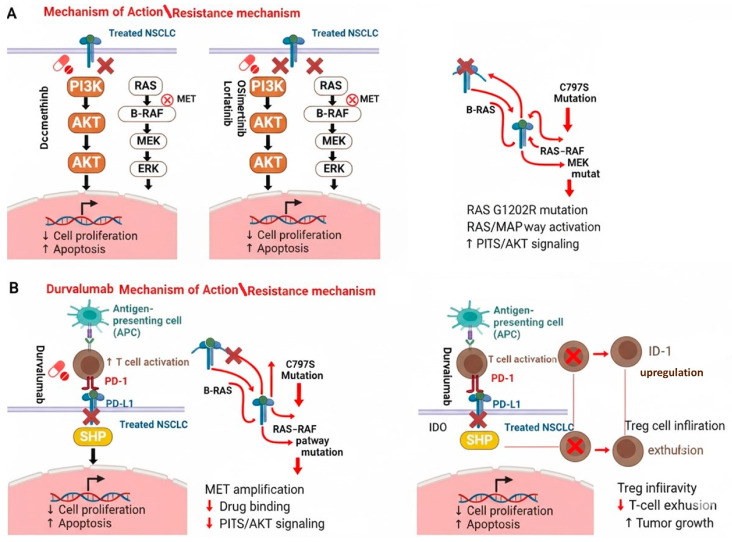
**Mechanism of action and resistance of TKIs.** PI3K-AKT, RAS, and SHP are essential TKIs drug target pathways. Human malignancies frequently exhibit deregulation of these pathways, which are prominent mediators of cell cycle development, proliferation, and apoptosis/cell survival. (**A**) Mechanism of action, dacomitinib blocks the PI3K/AKT pathway and thus promotes cell apoptosis, lorlatinib and osimertinib prevents both PI3K and RAS pathways, and hence enhances cell apoptosis. and (**B**) Durvalumab blocks the SHP pathway, and thus prevents cell survival. The resistance mechanisms are shown on the right-hand side.

Around the globe, dacomitinib is used as a first-line treatment for patients diagnosed with NSCLC metastasis. A conducted study by Nishio et al. claimed that dacomitinib improves the progression of free survival in patients, with no serious adverse events [6]. This suggests the ability of dacomitinib to act as an effective and safe first-line treatment for patients with EGFR-positive advanced NSCLC. The mechanism of action of dacomitinib in NSCLC is summarized in Figure 2A.

Despite its proven effectiveness, dacomitinib may still have a few adverse effects, though not all of these adverse effects will necessarily occur, though they might require medical care if they do develop. These adverse effects depend on the duration of treatment, the presence of inflammations such as stomatitis and dermatitis, and the presence of other symptoms, including diarrhea, chest pain, weight loss, loss of appetite, hair loss, coughing, sleep issues, vomiting, and swollen or red eyes.

Several studies have evaluated the role of dacomitinib in phases I, II, and III. These are listed in Table 1. There is also a newly completed phase IV clinical trial (n = 115) “Dacomitinib for the Treatment of Patients in India with Metastatic Non-Small Cell Lung Cancer with EGFR Activating Mutations” (NCT04511533), conducted by Pfizer, claiming that dacomitinib is effective and safe, with no serious adverse effects.

### 3.2. Lorlatinib

Larlatinib is an orally effective drug that regulates ALK and ROS1 kinases, it has been developed by Pfizer for ALK-positive NSCLC treatment [13]. Generally, ALK is a naturally occurring endogenous tyrosine kinase receptor that stimulates different kinds of neurons in the nervous system and is crucial for brain development. Larlatinib is a member of the third generation of ALK-TKI and shows extensive effectiveness against ALK-resistance mutations developed during therapies employing first-generation (crizotinib) or second-generation ALK inhibitors (alectinib, ceritinib) [14]. Moreover, lorlatinib has shown in vitro efficacy against a variety of ALK enzyme mutations, including several found in tumors where the clinical condition was worsening while using crizotinib and other ALK inhibitors [15]. Furthermore, lorlatinib penetrates the blood–brain barrier, and can also treat developing or worsening brain metastases [16]. In in vivo models, lorlatinib’s overall anticancer effect seems to depend on the dose and is associated with the suppression of ALK phosphorylation [17]. The mechanism of action of lorlatinib in NSCLC is summarized in Figure 2A.

Larlatinib was first approved in Japan in September 2018, followed by the United States in November 2018, based on the findings of phase I/II studies [18]. The European Medicines Agency (EMA) later authorized larlatinib in 2019 for the treatment of a selected group of patients with advanced ALK-positive NSCLC who had already received other treatments. In 2022, the approval was further extended to cover lorlatinib as a first-line treatment option for advanced ALK-positive NSCLC [19]. A multicentric phase I/II study (NCT01970865) showed strong antitumor efficacy of lorlatinib, following the failure of prior ALK inhibitor therapy, including in patients who had been treated with either first- or second-generation ALK TKIs, or both [20,21].

Lorlatinib is highly effective in the treatment of advanced NSCLC. The adverse effects of this agent include hypertriglyceridemia, hypercholesterolemia, central nerve system effects (speech disorder, depression, cognitive disorder), peripheral neuropathy, overweight edema and gastrointestinal effects. Moreover, atrioventricular block might occur due to an excessive increase in lipase. However, there is a slim chance of long-term withdrawal based on adverse effects, though the majority of them are temporary, and therefore, lorlatinib has an exceptional safety profile compared to other ALK TKIs medications [19].

Several Phase I, II, and III clinical studies have evaluated the efficacy of larlatinib in NSCLC patients. These are summarized in Table 2. There are also two completed Phase IV clinical trials, “Lorlatinib in ALK Inhibitor Treated Unresectable Advanced/Recurrent ALK-Positive Non-Small Cell Lung Cancer Patients in India” (NCT04541706: n = 100) and “Study of Loralatinib in People with ALK-positive Non-small Cell Lung Cancer” (NCT04362072: n = 71), conducted by Pfizer, to explore the efficacy and adverse effects of lorlatinib as a therapeutic agent for NSCLC. These studies reported that lorlatinib was effective and safer for advanced NSCLC.

### 3.3. Durvalumab

Durvalumab is an immune-target therapy developed by Medimmune/AstraZeneca that produces an antitumor antibody to stimulate immune-mediated antitumor responses via activating immune cells [28]. Durvalumab is a human immunoglobulin G1 kappa (IgG1k) monoclonal antibody that exclusively inhibits the interaction of human PD-L1 with programmed cell death 1 (PD-1) and cluster differentiation (CD80) [29,37]. This stimulates anti-cancer immune responses, T-cell proliferation, and T-cell activity [29]. Furthermore, it is designed to prevent cellular cytotoxicity reactions that are dependent on antibodies. Reductions in tumor growth are linked to durvalumab-induced PD-L1 inhibition in co-engrafted human cancer and immune cell xenograft mouse models [26]. To improve the effectiveness of PD-L1 blockade, it may be combined with other chemotherapeutic agents. The mechanism of action in NSCLC is summarized in Figure 2B. Durvalumab, used alone [33] or in conjunction with chemotherapy [34], has shown antitumor effects in individuals with NSCLS during early clinical trials. Likewise, a study conducted by Rothschild et al. reported that stage III NSCLC patients who received durvalumab alongside neoadjuvant chemotherapy showed good results and manageable adverse effects [35].

In 2018, durvalumab was approved by the FDA for stage III NSCLC. Moreover, durvalumab sequential monotherapy significantly improved progression-free survival in patients with unresectable stage III NSCLC who did not progress after platinum-based chemoradiation therapy, according to a planned interim analysis of a randomized, double-blind, placebo-controlled, phase III PACIFIC trial (NCT02125461) [36]. An update on PACIFIC trials conducted by Faivre-Finn et al. demonstrated that the standard-of-care PACIFIC combination with durvalumab after chemoradiotherapy indicates sustained overall survival (OS) benefit with persistent progression-free survival (PFS), as determined by preliminary findings from PACIFIC [38].

Although durvalumab has expanded utilization as an effective therapeutic target for advanced stages of NSCLC, using durvalumab for advanced stages of NSCLC may present some limitations. These adverse effects include shortness of breath, coughing, upper respiratory tract infections, inflammation of the lungs, chest pain, and fatigue. However, these adverse effects are manageable and easy to resolve with direct healthcare, which allows a faster response to any medical adverse reaction.

Multiple studies have investigated the role of durvalumab in phase II and III clinical trials. These are summarized in Table 3. There is also a phase IV clinical trial conducted by AstraZeneca that evaluated the role of durvalumab in advanced NSCLC: “Study to Access Safety of Durvalumab in Indian Adult Patients with Locally Advanced NSCLC” (NCT04416633: n = 100).

### 3.4. Osimertinib

Osimertinib (developed by AstraZeneca, UK, Cambridge) is a third-generation EFGR-TKI that particularly targets activating mutations of EGFR and the resistance of T790M mutations, a barrier mutation that enhances EGFR’s tolerance of ATP in its ATP-binding domain, thus effectively inhibiting first- and second-generation TKIs from attaching to EGFR ATP-binding sites [44]. Osimertinib acts by forming a covalent bond with the C797 residue at the ATP-binding site of mutant EGFR. In contrast to both first- and second-generation EGFR-TKIs, osimertinib exhibits greater efficacy towards T790M mutants in vitro, with low off-target effects, resulting in fewer adverse outcomes generally associated with the blockage of wild-type EGFR [45]. The mechanism of action in NSCLC is summarized in Figure 2A.

Initially, osimertinib was exclusively authorized for use in individuals with EGFR T790M-positive mutations whose NSCLC was advancing after EGFR TKI treatment [46]. Subsequently, FLAURA research revealed a notable improvement in both OS and PFS with osimertinib when compared to first-generation EGFR TKI therapy [47]. Nowadays, major healthcare organizations have authorized osimertinib as the sole third-generation EGFR-TKI for the management of T790M-positive patients who have made progress with first- or second-generation EGFR-TKIs. Furthermore, osimertinib received approval in 2018 as the primary therapeutic medication for advanced EFGR-mutated NSCLC [48].

Although osimertinib is recognized as one of the primary therapies in advanced NCSCLC, patients invariably develop secondary resistance to osimertinib, despite its strong clinical efficacy. This represents a key difficulty, because there are currently limited post-osimertinib pharmacological alternatives available. Moreover, the drug may have certain adverse effects in addition to its anticipated advantages. Although not all of these adverse reactions may be seen in all patients, if they do present, medical attention is required to treat/minimize these adverse reactions. These adverse reactions include swelling or inflammation of the mouth, back pain, constipation, nausea, and skin rush.

A summary of clinical trials that have evaluated the role of osimertinib in NSCLC in phase II and III clinical trials is presented in Table 4. A recently completed Phase IV clinical trial (NCT05219162; n = 182) conducted by AstraZeneca investigated the role of osimertinib in NSCLC: “Real-World Study on Gene Profile in Patients with Advanced NSCLC Who Progressed on First-Line Osimertinib Therapy (GPS)”.

## 4. Antibody-Drug Conjugates in Non-Small Cell Lung Cancer

The treatment landscape for non-small cell lung cancer (NSCLC) is being reshaped by antibody-drug conjugates (ADCs), which merge the precision of monoclonal antibodies with the destructive potential of cytotoxic agents. These sophisticated molecules are engineered to selectively target tumor cells, thereby widening the therapeutic window and reducing systemic toxicity. Each ADC comprises three essential components: a monoclonal antibody designed to recognize specific tumor antigens, a chemical linker that ensures stability in circulation, and a potent cytotoxic payload. The drug-to-antibody ratio (DAR) plays a crucial role in determining both efficacy and safety profiles. A significant feature of many ADCs is the bystander effect, wherein the released payload can penetrate nearby cells, targeting heterogeneous tumor populations that may not express the antigen [54]. Significant clinical progress has been achieved with HER2-directed agents. Trastuzumab deruxtecan (T-DXd) gained regulatory approval for HER2-mutant NSCLC based on results from the DESTINY-Lung trials, which reported a 55% objective response rate and 8.2 months median progression-free survival [55]. Earlier efforts with trastuzumab emtansine (T-DM1), however, demonstrated more limited clinical activity in this setting [56].

Emerging targets beyond HER2 show considerable promise. Datopotamab deruxtecan (Dato-DXd), which targets TROP2, improved progression-free survival compared to docetaxel in the TROPION-Lung01 study, though overall survival data are still maturing [57]. Similarly, patritumab deruxtecan (HER3-DXd) achieved a 29.8% response rate in EGFR-mutant patients who had progressed on prior tyrosine kinase inhibitors and chemotherapy [58]. Telisotuzumab vedotin, targeting c-MET, has also demonstrated encouraging anti-tumor activity in selected patient subgroups. Despite these advances, several challenges require attention. Management of unique toxicities, particularly interstitial lung disease associated with T-DXd, demands careful monitoring and prompt intervention. The development of reliable predictive biomarkers beyond antigen expression levels remains a critical need for optimizing patient selection. Additionally, understanding resistance mechanisms and determining optimal sequencing strategies represent important areas for future research. ADCs have undoubtedly expanded therapeutic options for NSCLC patients. Ongoing research efforts continue to refine their clinical application, with a focus on enhancing efficacy while managing treatment-related adverse events.

## 5. Anti-Angiogenic Agents in the Management of Non-Small Cell Lung Cancer

Angiogenesis, the formation of new blood vessels, is a critical process for tumor growth and metastasis. Vascular endothelial growth factor (VEGF) and its receptors are key mediators of this process. Anti-angiogenic agents, such as the monoclonal antibody bevacizumab which targets VEGF-A, inhibit this pathway, thereby suppressing tumor blood supply and promoting normalization of the remaining vasculature. This normalization can reduce intratumoral pressure and improve the delivery of concurrently administered chemotherapeutic or immunotherapeutic agents. The most substantiated evidence for anti-angiogenic therapy in NSCLC comes from the IMpower150 trial [59]. This phase III study evaluated a quadruple regimen consisting of atezolizumab (an anti-PD-L1 antibody), bevacizumab (anti-VEGF), carboplatin, and paclitaxel (ABCP) compared to bevacizumab plus chemotherapy (BCP) in patients with metastatic non-squamous NSCLC without EGFR or ALK alterations. The ABCP regimen demonstrated a statistically significant improvement in overall survival (OS) compared to BCP, with a median OS of 19.5 months versus 14.7 months (HR 0.80; 95% CI, 0.67–0.95) [59]. Exploratory analyses indicated that the survival benefit was most evident in patients with high PD-L1 expression and those with liver metastases. Notably, the trial design allowed for continuous atezolizumab treatment until loss of clinical benefit, differing from the fixed-duration approach used in other immunotherapy trials. This regimen provides an important first-line option, particularly for patients with high tumor burden or aggressive disease phenotypes where rapid disease control is paramount.

The combination of an anti-angiogenic agent with immunotherapy is thought to work synergistically; bevacizumab may reverse VEGF-mediated immunosuppression in the tumor microenvironment, potentially enhancing the efficacy of immune checkpoint inhibition. Despite its benefits, the use of bevacizumab is restricted to patients with non-squamous histology due to an elevated risk of life-threatening hemoptysis in patients with squamous cell carcinoma. Furthermore, anti-angiogenic agents are associated with a distinct toxicity profile, including hypertension, proteinuria, bleeding events, and impaired wound healing, which necessitates careful patient selection and monitoring. The IMpower150 trial also showed that the benefit was less clear in the PD-L1 negative population, highlighting that not all patients derive equal benefit from this intensive and costly combination [59]. Anti-angiogenic therapy, exemplified by bevacizumab in the IMpower150 regimen, remains a valuable component of combination therapy for a subset of patients with advanced non-squamous NSCLC. Its role has been refined rather than replaced by the advent of immunotherapy, finding a niche in synergistic quadruple regimens. Future research directions include identifying predictive biomarkers beyond histology to better select patients who will benefit most from anti-angiogenic combinations. Additionally, investigating novel anti-angiogenic agents and their combinations with newer immunotherapeutic strategies, such as bispecific antibodies or antibody-drug conjugates, may further expand the utility of this treatment modality.

## 6. Chimeric Antigen Receptor (CAR)-T-Cell Therapy for NSCLC

Chimeric antigen receptor (CAR)-T-cell therapy is a promising and novel strategy for the treatment of non-small-cell lung cancer (NSCLC). While anti-CD19 CAR-T cells have been approved for hematological malignancies, their application to solid tumors like NSCLC is still in development. The therapy involves genetically engineering T cells to express a CAR that specifically recognizes and binds to tumor-associated antigens (TAAs). Despite their potential, the clinical application of CAR-T cells in NSCLC faces significant challenges. These include the scarcity of truly tumor-specific antigens, an immunosuppressive tumor microenvironment, poor infiltration of CAR-T cells into tumor tissue, and the risk of tumor antigen escape. Additionally, toxicities such as cytokine release syndrome (CRS), neurological toxicity, and on-target/off-tumor effects—where CAR-T cells attack healthy tissue expressing the same antigen—remain major safety concerns [60]. Current research is focused on identifying suitable antigens to target in NSCLC. The most commonly targeted antigens in clinical trials include EGFR, mesothelin (MSLN), mucin 1 (MUC1), prostate stem cell antigen (PSCA), carcinoembryonic antigen (CEA), and programmed death-ligand 1 (PD-L1).

Several clinical trials have explored these targets. Anti-EGFR CAR-T cells have shown specific cytolytic activity against EGFR-positive tumor cells in preclinical studies. A phase I trial (NCT04153799) evaluating anti-EGFR CAR-T cells in advanced NSCLC patients reported that two out of eleven patients achieved a partial response, while five had stable disease for eight months. Anti-MSLN CAR-T cells are also being tested, and preliminary results demonstrate the rationale for their use, though some trials have been terminated due to slow patient enrollment (NCT01583686). For antigens like MUC1 and PSCA, ongoing studies are assessing the safety and efficacy of CAR-T cells for advanced solid tumors, including NSCLC (NCT02587689 and NCT03198052) [60]. A preclinical model indicated that a third-generation anti-PSCA CAR-T cell delayed tumor development. Furthermore, anti-CEA CAR-T cell therapies are in phase I trials to evaluate their safety and efficacy in lung cancer patients who are CEA-positive (NCT02349724 and NCT04348643). Researchers are also investigating anti-PD-L1 CAR-T cell therapy as a novel strategy, as PD-L1 is expressed on both tumor and stromal cells. Early phase I studies are underway to determine the safety and engraftment potential of these cells (NCT03060343, NCT03330834). Similarly, anti-ROR1 CAR-T cells are being tested for their anti-tumor effects in advanced ROR1-positive, stage IV NSCLC patients (NCT02706392). The article also notes that while a trial for anti-HER2 CAR-T cells was withdrawn due to safety considerations, this antigen remains a potential therapeutic target. While CAR-T therapy for NSCLC is still in its early stages, it has yielded promising preliminary results in both preclinical and clinical settings. Continued research is essential to overcome current challenges and realize its full therapeutic potential.

## 7. Bispecific Antibodies for NSCLC

Bispecific antibodies represent a transformative and highly relevant innovation in treating NSCLC. These new therapies effectively highlight a paradigm shift, moving beyond the single-target approach of conventional treatments [61]. A particular emphasis is placed on the transformative impact of amivantamab, an epidermal growth factor receptor (EGFR)/mesenchymal–epithelial transition factor (MET)-targeting antibody [62]. This therapy has revolutionized the treatment landscape for patients with EGFR exon 20 insertion mutations—a patient population that has historically shown poor response to standard tyrosine kinase inhibitors. The inclusion of other emerging agents, such as zenocutuzumab for tumors with neuregulin 1 fusions, further illustrates the expanding utility of bispecific antibodies in addressing specific, previously untreatable oncogenic drivers [62]. By synthesizing key data and recent approvals, it is clear that these agents are addressing significant unmet clinical needs and shaping future treatment strategies. Beyond the discussion of clinical efficacy, the focus is on the “real-world” aspects of integrating these novel drugs. A much-needed framework is provided for managing the unique spectrum of on-target toxicities, including dermatologic side effects and infusion-related reactions. The practical guidance offered is invaluable, ensuring that the promise of these therapies can be realized in everyday practice without compromising patient safety and well-being. Furthermore, consideration of broader patient-centered issues, such as quality-of-life outcomes and financial toxicity, sets this review apart [63]. The exploration of subcutaneous formulations as a means to improve patient adherence and convenience is a forward-thinking and crucial point for therapies that require long-term administration. This thoughtful approach ensures the content is not just a summary of clinical data, but a pragmatic tool for enhancing patient care.

The clinical application of bispecific antibodies is a significant and timely contribution to the field of thoracic oncology. It provides a balanced and thorough account of these therapies, celebrating their clinical successes while offering a realistic perspective on the challenges of their implementation. The strength of this work lies in its ability to bridge the gap between academic innovation and practical, patient-focused care. It will serve as an essential reference for clinicians as the pipeline of bispecific antibodies continues to expand and these treatments become more integrated into standard practice.

## 8. Alternative Medicine

While some investigations have explored the role of traditional and alternative medicine in non-small-cell lung cancer (NSCLC) management, it is crucial to maintain a cautious perspective and avoid overstating the clinical value of these approaches when used alongside established pharmacological treatments. The current evidence base, while showing some promise, requires careful interpretation.

Traditional Chinese Medicine (TCM) has been investigated for potential benefits, with some reports suggesting positive effects for chronic pain when used either alone or as a supplementary therapy [64,65,66]. A study by Jiang et al. indicated that patients who received TCM alone experienced effects on time to progression and overall survival that were reportedly similar to those of chemotherapy [67]. Additionally, a separate study by Li et al. suggested that prophylactic TCM might potentially help reduce the incidence of grade 3 skin toxicity [68]. There are also reports that TCM, whether as a stand-alone therapy or in combination with conventional treatments like immunotherapy, chemotherapy, radiotherapy, targeted therapy, and surgery, has been associated with improved patient outcomes for NSCLC.

These findings, however, should be viewed with a critical eye. While they hint at areas for further exploration, they do not yet definitively establish the role of TCM within the standard of care. To confirm these potential benefits, more rigorous, large-scale clinical trials are needed to validate its safety and effectiveness in improving patient outcomes [69].

## 9. Emerging Treatments

Fascinating novel molecules have shown potential in the ongoing fight towards NSCLC. Over many years, TKIs, which target oncogenic driver mutations, have improved in efficacy and addressed drug resistance [70]. Moreover, immune checkpoint inhibiting drugs remain vital for treating NSCLCs that lack particular identifiable genetic alterations [71]. Likewise, a study conducted by Hayashi et al. reported that a combination of liquid immune components could pinpoint individuals who are anticipated to be responsive to PD-1/PD-L1 inhibition due to terminal depletion of antitumor immunity, and therefore, this combination might be able to better predict patient response to NSCLC [72]. Another recent study reported that trophoblast cell surface antigen 2 (TROP2) expression performs a crucial role in the main resistance to PD-L1 inhibition in NSCLC, and this could help in identifying patients with NSCLC who might benefit from a combination of immunotherapy and an anti-TROP2 drug [73].

Cancer-associated thrombosis (CAT) is a significant thrombotic event in cancer patients [74]. Recent research has investigated the association between thromboembolic events and immune checkpoint inhibitor therapies in NSCLC patients, and revealed that the probability of thrombosis among NSCLC is not insignificant [75]. This detection could provide a potential insight as regards developing a novel NSCLC therapy approach, as well as its diagnostic instruments, and subsequently enhance NSCLC patient outcomes. Therefore, more investigation is warranted to explore the key mechanism factor(s) underlying the association between cancer, hemostasis, and immune response in the environment of immune checkpoint inhibitor treatments. Nevertheless, discovering predictive biomarkers to customize therapies, overcome resistance, reduce costs, and offer appropriate cancer treatment availability remains an ongoing challenge.

## 10. Conclusions

Effective therapy for non-small-cell lung cancer (NSCLC) remains a significant challenge, despite the pivotal role of immune checkpoint inhibitors and molecular targeted therapies. The limitations of these treatments, particularly the development of drug resistance, necessitate a focus on several key research priorities to enhance patient outcomes. A central research priority is to understand and address the mechanisms of both primary and acquired drug resistance. This requires investigating why some patients fail to respond to initial therapies and why others eventually relapse. The treatment landscape is being rapidly reshaped by novel therapeutic modalities. Antibody-drug conjugates (ADCs) have emerged as a powerful class of drugs, merging the precision of monoclonal antibodies with the potency of cytotoxic payloads to target specific tumor antigens like HER2, TROP2, and HER3, offering new options for heavily pre-treated patients. Similarly, anti-angiogenic agents continue to play a crucial role, particularly in synergistic combinations with immunotherapy and chemotherapy, as evidenced by regimens like atezolizumab–bevacizumab–carboplatin–paclitaxel, which improve survival in selected patient populations. Beyond these, cellular therapies, such as CAR-T cells targeting antigens like EGFR, MSLN, and PD-L1, represent a frontier in personalized medicine, though their application in solid tumors like NSCLC requires overcoming significant challenges related to the tumor microenvironment and toxicity. Furthermore, bispecific antibodies are demonstrating transformative potential by engaging multiple targets simultaneously; amivantamab, targeting EGFR and MET, has revolutionized care for patients with EGFR exon 20 insertion mutations, highlighting a shift towards addressing previously untreatable oncogenic drivers. Advancing personalized medicine in NSCLC depends on identifying more reliable predictive biomarkers for all these therapies. While PD-L1 expression and genetic mutations are useful indicators, they do not consistently predict treatment response. Future research should aim to discover and validate a broader range of biomarkers to allow for more precise patient selection, ensuring that individuals receive the most appropriate therapy while minimizing unnecessary exposure to ineffective treatments.

Finally, a key research priority is the seamless integration of these novel therapeutic modalities—including ADCs, anti-angiogenics, cellular therapies, and bispecific antibodies—into existing and new treatment regimens. This includes exploring their use in combination with each other and with conventional strategies like chemotherapy, radiotherapy, and surgery. By thoughtfully combining these innovative approaches, clinicians can create more comprehensive and personalized treatment plans, ultimately leading to more effective NSCLC management and improved patient outcomes.

## Figures and Tables

**Table 1:** Overview of Dacomitinib in clinical trial phases I, II, and III.

N	Authors/Reference	Clinical Trails	Phase of the Studies	Number of Samples
1	Jung H. et al. [7]	(NCT04339829)	Phase II	30
2	Iwasaku M. et al. [8]	(jRCTs071190015)	Phase II	41
3	Tan W. et al. [9]	(NCT01858389)	Phase II	41
4	Mok T. et al. [10]	(NCT01774721)	Phase III	227
5	Pu X. et al. [11]	(NCT01774721)	Phase III	227
6	Cheng Y. et al. [12]	(NCT01774721)	Phase III	346

**Table 2:** Overview of Lorlatinib in clinical trial phases I, II, and III.

N	Authors/Reference	Clinical Trails	Phase of the Studies	Number of Samples
1	Goldsmith K. et al. [22]	(NCT03107988)	Phase I	65
2	Solomon B. et al. [21]	(NCT01970865)	Phase I & II	364
3	Lu S. et al. [23]	(NCT03909971)	Phase II	109
4	Felip E et al. [24]	(NCT01970865)	Phase II	139
5	Ou S. et al. [25]	(NCT01970865)	Phase I & II	364
6	Solomon B. et al. [27]	(NCT03052608)	Phase III	296

**Table 3:** Overview of Durvalumab in clinical trials- phase II and III.

N	Authors/Reference	Clinical Trails	Phase of the Studies	Number of Samples
1	Besse B. et al. [39]	(NCT03334617)	Phase II	268
2	Garassino M. et al. [40]	(NCT03693300)	Phase II	117
3	Reck M. et al. [41]	(NCT05925530)	Phase II	140
4	Schoenfeld J. et al. [42]	(NCT02888743)	Phase II	90
5	Johnson M. et al. [33]	(NCT03164616)	Phase III	1013
6	Ruysscher D. et al. [43]	(NCT04026412)	Phase III	888

**Table 4:** Overview of Osimertinib in clinical trials- II and III.

N	Authors/Reference	Clinical Trails	Phase of the Studies	Number of Samples
1	Okuma Y. et al. [49]	(jRCTs071200002)	Phase II	40
2	Herbst R. et al. [50]	(NCT02511106)	Phase III	682
3	Lu S. et al. [51]	(NCT03521154)	Phase III	216
4	Planchard D. et al. [52]	(NCT04035486)	Phase III	557
5	Remon J. et al. [53]	(NCT02856893)	Phase III	156

## References

[1] Shirley M. (2018). Dacomitinib: First Global Approval. Drugs.

[2] Passaro A., Mok T., Peters S., Popat S., Ahn M.J., de Marinis F. (2021). Recent Advances on the Role of EGFR Tyrosine Kinase Inhibitors in the Management of NSCLC with Uncommon, Non Exon 20 Insertions, EGFR Mutations. J. Thorac. Oncol..

[3] Wu S.G., Yu C.J., Yang J.C., Shih J.Y. (2020). The effectiveness of afatinib in patients with lung adenocarcinoma harboring complex epidermal growth factor receptor mutation. Ther. Adv. Med. Oncol..

[4] Niu Z.X., Wang Y.T., Lu N., Sun J.F., Nie P., Herdewijn P. (2023). Advances of clinically approved small-molecule drugs for the treatment of non-small cell lung cancer. Eur. J. Med. Chem..

[5] Shah R.R., Shah D.R. (2019). Safety and Tolerability of Epidermal Growth Factor Receptor (EGFR) Tyrosine Kinase Inhibitors in Oncology. Drug Saf..

[6] Nishio M., Kato T., Niho S., Yamamoto N., Takahashi T., Nogami N., Kaneda H., Fujita Y., Wilner K., Yoshida M. (2020). Safety and efficacy of first-line dacomitinib in Japanese patients with advanced non-small cell lung cancer. Cancer Sci..

[7] Jung H.A., Park S., Lee S.H., Ahn J.S., Ahn M.J., Sun J.M. (2023). Dacomitinib in EGFR-mutant non-small-cell lung cancer with brain metastasis: A single-arm, phase II study. ESMO Open.

[8] Iwasaku M., Uchino J., Yamada T., Chihara Y., Shimamoto T., Tamiya N., Kaneko Y., Kiyomi F., Takayama K. (2019). Rationale and design of a phase II study to evaluate prophylactic treatment of dacomitinib-induced dermatologic adverse events in epidermal growth factor receptor-mutated advanced non-small cell lung cancer (SPIRAL-Daco study). Transl. Lung Cancer Res..

[9] Tan W., Giri N., Quinn S., Wilner K., Parivar K. (2020). Evaluation of the potential effect of dacomitinib, an EGFR tyrosine kinase inhibitor, on ECG parameters in patients with advanced non-small cell lung cancer. Investig. New Drugs.

[10] Mok T.S., Cheng Y., Zhou X., Lee K.H., Nakagawa K., Niho S., Chawla A., Rosell R., Corral J., Migliorino M.R. (2021). Updated Overall Survival in a Randomized Study Comparing Dacomitinib with Gefitinib as First-Line Treatment in Patients with Advanced Non-Small-Cell Lung Cancer and EGFR-Activating Mutations. Drugs.

[11] Pu X., Li J., Zhang B., Zhang J., TS K.M., Nakagawa K., Rosell R., Cheng Y., Zhou X., Miglorino M.R. (2024). Efficacy in patients with EGFR-positive non-small-cell lung cancer treated with dacomitinib who had skin adverse events: Post hoc analyses from ARCHER 1050. Future Oncol..

[12] Cheng Y., Mok T.S., Zhou X., Lu S., Zhou Q., Zhou J., Du Y., Yu P., Liu X., Hu C. (2021). Safety and efficacy of first-line dacomitinib in Asian patients with EGFR mutation-positive non-small cell lung cancer: Results from a randomized, open-label, phase 3 trial (ARCHER 1050). Lung Cancer.

[13] Choo J.R., Soo R.A. (2020). Lorlatinib for the treatment of ALK-positive metastatic non-small cell lung cancer. Expert. Rev. Anticancer. Ther..

[14] Goto Y., Shukuya T., Murata A., Kikkawa H., Emir B., Wiltshire R., Miura S. (2023). Real-world therapeutic effectiveness of lorlatinib after alectinib in Japanese patients with ALK-positive non-small-cell lung cancer. Cancer Sci..

[15] Solomon B.J., Liu G., Felip E., Mok T.S.K., Soo R.A., Mazieres J., Shaw A.T., de Marinis F., Goto Y., Wu Y.L. (2024). Lorlatinib Versus Crizotinib in Patients with Advanced ALK-Positive Non-Small Cell Lung Cancer: 5-Year Outcomes from the Phase III CROWN Study. J. Clin. Oncol..

[16] Murakami Y., Kawashima Y., Chiba S., Hara S., Yamazaki Y., Doman T., Saito S., Odaka T., Ogasawara T., Shimizu H. (2024). Successful application of lorlatinib in a 23-year-old patient with anaplastic lymphoma kinase (ALK)-positive lung cancer and multiple brain metastases. Cancer Rep..

[17] Taniguchi H., Akagi K., Dotsu Y., Yamada T., Ono S., Imamura E., Gyotoku H., Takemoto S., Yamaguchi H., Sen T. (2023). Pan-HER inhibitors overcome lorlatinib resistance caused by NRG1/HER3 activation in ALK-rearranged lung cancer. Cancer Sci..

[18] Syed Y.Y. (2019). Lorlatinib: First Global Approval. Drugs.

[19] Naik J., Beavers N., Nilsson F.O.L., Iadeluca L., Lowry C. (2023). Cost—Effectiveness of Lorlatinib in First-Line Treatment of Adult Patients with Anaplastic Lymphoma Kinase (ALK)—Positive Non—Small—Cell Lung Cancer in Sweden. Appl. Health Econ. Health Policy.

[20] Shaw A.T., Felip E., Bauer T.M., Besse B., Navarro A., Postel-Vinay S., Gainor J.F., Johnson M., Dietrich J., James L.P. (2017). Lorlatinib in non-small-cell lung cancer with ALK or ROS1 rearrangement: An international, multicentre, open-label, single-arm first-in-man phase 1 trial. Lancet Oncol..

[21] Solomon B.J., Besse B., Bauer T.M., Felip E., Soo R.A., Camidge D.R., Chiari R., Bearz A., Lin C.C., Gadgeel S.M. (2018). Lorlatinib in patients with ALK-positive non-small-cell lung cancer: Results from a global phase 2 study. Lancet Oncol..

[22] Goldsmith K.C., Park J.R., Kayser K., Malvar J., Chi Y.Y., Groshen S.G., Villablanca J.G., Krytska K., Lai L.M., Acharya P.T. (2023). Lorlatinib with or without chemotherapy in ALK-driven refractory/relapsed neuroblastoma: Phase 1 trial results. Nat. Med..

[23] Lu S., Zhou Q., Liu X., Du Y., Fan Y., Cheng Y., Fang J., Lu Y., Huang C., Zhou J. (2022). Lorlatinib for Previously Treated ALK-Positive Advanced NSCLC: Primary Efficacy and Safety from a Phase 2 Study in People’s Republic of China. J. Thorac. Oncol..

[24] Felip E., Shaw A.T., Bearz A., Camidge D.R., Solomon B.J., Bauman J.R., Bauer T.M., Peters S., Toffalorio F., Abbattista A. (2021). Intracranial and extracranial efficacy of lorlatinib in patients with ALK-positive non-small-cell lung cancer previously treated with second-generation ALK TKIs. Ann. Oncol..

[25] Ou S.I., Solomon B.J., Shaw A.T., Gadgeel S.M., Besse B., Soo R.A., Abbattista A., Toffalorio F., Wiltshire R., Bearz A. (2022). Continuation of Lorlatinib in ALK-Positive NSCLC Beyond Progressive Disease. J. Thorac. Oncol..

[26] Stewart R., Morrow M., Hammond S.A., Mulgrew K., Marcus D., Poon E., Watkins A., Mullins S., Chodorge M., Andrews J. (2015). Identification and Characterization of MEDI4736, an Antagonistic Anti-PD-L1 Monoclonal Antibody. Cancer Immunol. Res..

[27] Solomon B.J., Bauer T.M., Mok T.S.K., Liu G., Mazieres J., de Marinis F., Goto Y., Kim D.W., Wu Y.L., Jassem J. (2023). Efficacy and safety of first-line lorlatinib versus crizotinib in patients with advanced, ALK-positive non-small-cell lung cancer: Updated analysis of data from the phase 3, randomised, open-label CROWN study. Lancet Respir. Med..

[28] Al-Salama Z.T. (2021). Durvalumab: A Review in Extensive-Stage SCLC. Target. Oncol..

[29] AstraZeneca (2021). MFINZI (Durvalumab): EU Summary of Product Characteristics. https://www.ema.europa.eu/en/documents/product-information/imfinzi-epar-product-information_en.pdf.

[30] Banna G.L., Cantale O., Bersanelli M., Del Re M., Friedlaender A., Cortellini A., Addeo A. (2020). Are anti-PD1 and anti-PD-L1 alike? The non-small-cell lung cancer paradigm. Oncol. Rev..

[31] Ando K., Manabe R., Kishino Y., Kusumoto S., Yamaoka T., Tanaka A., Ohmori T., Ohnishi T., Sagara H. (2020). Comparative Efficacy and Safety of Anti-PD-1/PD-L1 Immune Checkpoint Inhibitors for Refractory or Relapsed Advanced Non-Small-Cell Lung Cancer—A Systematic Review and Network Meta-Analysis. Cancers.

[32] Ding J., Liu Z., Ning J., Sun N. (2025). Safety of PD-L1 inhibitors versus PD-1 inhibitors in the treatment of lung cancer: A systematic review and network meta-analysis. Expert. Rev. Anticancer. Ther..

[33] Johnson M.L., Cho B.C., Luft A., Alatorre-Alexander J., Geater S.L., Laktionov K., Kim S.W., Ursol G., Hussein M., Lim F.L. (2023). Durvalumab with or Without Tremelimumab in Combination with Chemotherapy as First-Line Therapy for Metastatic Non-Small-Cell Lung Cancer: The Phase III POSEIDON Study. J. Clin. Oncol..

[34] Heymach J.V., Harpole D., Mitsudomi T., Taube J.M., Galffy G., Hochmair M., Winder T., Zukov R., Garbaos G., Gao S. (2023). Perioperative Durvalumab for Resectable Non-Small-Cell Lung Cancer. N. Engl. J. Med..

[35] Rothschild S.I., Zippelius A., Eboulet E.I., Savic Prince S., Betticher D., Bettini A., Früh M., Joerger M., Lardinois D., Gelpke H. (2021). SAKK 16/14: Durvalumab in Addition to Neoadjuvant Chemotherapy in Patients with Stage IIIA(N2) Non-Small-Cell Lung Cancer—A Multicenter Single-Arm Phase II Trial. J. Clin. Oncol..

[36] AstraZeneca IMFINZI™ (Durvalumab) Significantly Reduces the Risk of Disease Worsening or Death in the Phase III PACIFIC Trial for Stage III Unresectable Lung Cancer. https://www.astrazeneca.com/media-centre/press-releases/2017/imfinzi-significantly-reduces-the-risk-of-disease-worsening-or-death-in-the-phase-iii-pacific-trial-for-stage-iii-unresectable-lung-cancer-12052017.html#!.

[37] AstraZeneca IMFINZI^®®^ (Durvalumab) Injection, for Intravenous Use: US Prescribing Information. https://www.accessdata.fda.gov/drugsatfda_docs/label/2021/761069s029lbl.pdf.

[38] Faivre-Finn C., Vicente D., Kurata T., Planchard D., Paz-Ares L., Vansteenkiste J.F., Spigel D.R., Garassino M.C., Reck M., Senan S. (2021). Four-Year Survival with Durvalumab After Chemoradiotherapy in Stage III NSCLC—An Update from the PACIFIC Trial. J. Thorac. Oncol..

[39] Besse B., Pons-Tostivint E., Park K., Hartl S., Forde P.M., Hochmair M.J., Awad M.M., Thomas M., Goss G., Wheatley-Price P. (2024). Biomarker-directed targeted therapy plus durvalumab in advanced non-small-cell lung cancer: A phase 2 umbrella trial. Nat. Med..

[40] Garassino M.C., Mazieres J., Reck M., Chouaid C., Bischoff H., Reinmuth N., Cove-Smith L., Mansy T., Cortinovis D., Migliorino M.R. (2022). Durvalumab After Sequential Chemoradiotherapy in Stage III, Unresectable NSCLC: The Phase 2 PACIFIC-6 Trial. J. Thorac. Oncol..

[41] Reck M., Nadal E., Girard N., Filippi A.R., Martin L.W., Gay C.M., Petersen C., Gale D., Emeribe U.A., Georgoulia N. (2024). MDT-BRIDGE: Neoadjuvant Durvalumab Plus Chemotherapy Followed by Either Surgery and Adjuvant Durvalumab or Chemoradiotherapy and Consolidation Durvalumab in Resectable or Borderline-resectable Stage IIB-IIIB NSCLC. Clin. Lung Cancer.

[42] Schoenfeld J.D., Giobbie-Hurder A., Ranasinghe S., Kao K.Z., Lako A., Tsuji J., Liu Y., Brennick R.C., Gentzler R.D., Lee C. (2022). Durvalumab plus tremelimumab alone or in combination with low-dose or hypofractionated radiotherapy in metastatic non-small-cell lung cancer refractory to previous PD(L)-1 therapy: An open-label, multicentre, randomised, phase 2 trial. Lancet Oncol..

[43] De Ruysscher D., Ramalingam S., Urbanic J., Gerber D.E., Tan D.S.W., Cai J., Li A., Peters S. (2022). CheckMate 73L: A Phase 3 Study Comparing Nivolumab Plus Concurrent Chemoradiotherapy Followed by Nivolumab with or Without Ipilimumab Versus Concurrent Chemoradiotherapy Followed by Durvalumab for Previously Untreated, Locally Advanced Stage III Non-Small-Cell Lung Cancer. Clin. Lung Cancer.

[44] Leonetti A., Sharma S., Minari R., Perego P., Giovannetti E., Tiseo M. (2019). Resistance mechanisms to osimertinib in EGFR-mutated non-small cell lung cancer. Br. J. Cancer.

[45] Liu Q., Luo Y., Li Z., Chen C., Fang L. (2021). Structural modifications on indole and pyrimidine rings of osimertinib lead to high selectivity towards L858R/T790M double mutant enzyme and potent antitumor activity. Bioorg Med. Chem..

[46] Papadimitrakopoulou V.A., Mok T.S., Han J.Y., Ahn M.J., Delmonte A., Ramalingam S.S., Kim S.W., Shepherd F.A., Laskin J., He Y. (2020). Osimertinib versus platinum-pemetrexed for patients with EGFR T790M advanced NSCLC and progression on a prior EGFR-tyrosine kinase inhibitor: AURA3 overall survival analysis. Ann. Oncol..

[47] Ramalingam S.S., Vansteenkiste J., Planchard D., Cho B.C., Gray J.E., Ohe Y., Zhou C., Reungwetwattana T., Cheng Y., Chewaskulyong B. (2020). Overall Survival with Osimertinib in Untreated, EGFR-Mutated Advanced NSCLC. N. Engl. J. Med..

[48] Soria J.C., Ohe Y., Vansteenkiste J., Reungwetwattana T., Chewaskulyong B., Lee K.H., Dechaphunkul A., Imamura F., Nogami N., Kurata T. (2018). Osimertinib in Untreated EGFR-Mutated Advanced Non-Small-Cell Lung Cancer. N. Engl. J. Med..

[49] Okuma Y., Kubota K., Shimokawa M., Hashimoto K., Kawashima Y., Sakamoto T., Wakui H., Murakami S., Okishio K., Hayashihara K. (2024). First-Line Osimertinib for Previously Untreated Patients with NSCLC and Uncommon EGFR Mutations: The UNICORN Phase 2 Nonrandomized Clinical Trial. JAMA Oncol..

[50] Herbst R.S., Wu Y.L., John T., Grohe C., Majem M., Wang J., Kato T., Goldman J.W., Laktionov K., Kim S.W. (2023). Adjuvant Osimertinib for Resected EGFR-Mutated Stage IB-IIIA Non-Small-Cell Lung Cancer: Updated Results from the Phase III Randomized ADAURA Trial. J. Clin. Oncol..

[51] Lu S., Kato T., Dong X., Ahn M.J., Quang L.V., Soparattanapaisarn N., Inoue T., Wang C.L., Huang M., Yang J.C. (2024). Osimertinib after Chemoradiotherapy in Stage III EGFR-Mutated NSCLC. N. Engl. J. Med..

[52] Planchard D., Jänne P.A., Cheng Y., Yang J.C., Yanagitani N., Kim S.W., Sugawara S., Yu Y., Fan Y., Geater S.L. (2023). Osimertinib with or without Chemotherapy in EGFR-Mutated Advanced NSCLC. N. Engl. J. Med..

[53] Remon J., Besse B., Aix S.P., Callejo A., Al-Rabi K., Bernabe R., Greillier L., Majem M., Reguart N., Monnet I. (2024). Overall Survival from the EORTC LCG-1613 APPLE Trial of Osimertinib Versus Gefitinib Followed by Osimertinib in Advanced EGFR-Mutant Non-Small-Cell Lung Cancer. J. Clin. Oncol..

[54] Drago J.Z., Modi S., Chandarlapaty S. (2021). Unlocking the potential of antibody-drug conjugates for cancer therapy. Nat. Rev. Clin. Oncol..

[55] Li B.T., Smit E.F., Goto Y., Nakagawa K., Udagawa H., Mazières J., Nagasaka M., Bazhenova L., Saltos A.N., Felip E. (2022). Trastuzumab Deruxtecan in HER2-Mutant Non-Small-Cell Lung Cancer. N. Engl. J. Med..

[56] Li B.T., Shen R., Buonocore D., Olah Z.T., Ni A., Ginsberg M.S., Ulaner G.A., Offin M., Feldman D., Hembrough T. (2018). Ado-Trastuzumab Emtansine for Patients with HER2-Mutant Lung Cancers: Results from a Phase II Basket Trial. J. Clin. Oncol..

[57] Ahn M.J., Tanaka K., Paz-Ares L., Cornelissen R., Girard N., Pons-Tostivint E., Vicente Baz D., Sugawara S., Cobo M., Pérol M. (2025). Datopotamab Deruxtecan Versus Docetaxel for Previously Treated Advanced or Metastatic Non-Small Cell Lung Cancer: The Randomized, Open-Label Phase III TROPION-Lung01 Study. J. Clin. Oncol..

[58] Yu H.A., Goto Y., Hayashi H., Felip E., Chih-Hsin Yang J., Reck M., Yoh K., Lee S.H., Paz-Ares L., Besse B. (2023). HERTHENA-Lung01, a Phase II Trial of Patritumab Deruxtecan (HER3-DXd) in Epidermal Growth Factor Receptor-Mutated Non-Small-Cell Lung Cancer After Epidermal Growth Factor Receptor Tyrosine Kinase Inhibitor Therapy and Platinum-Based Chemotherapy. J. Clin. Oncol..

[59] Socinski M.A., Nishio M., Jotte R.M., Cappuzzo F., Orlandi F., Stroyakovskiy D., Nogami N., Rodríguez-Abreu D., Moro-Sibilot D., Thomas C.A. (2021). IMpower150 Final Overall Survival Analyses for Atezolizumab Plus Bevacizumab and Chemotherapy in First-Line Metastatic Nonsquamous NSCLC. J. Thorac. Oncol..

[60] Qu J., Mei Q., Chen L., Zhou J. (2021). Chimeric antigen receptor (CAR)-T-cell therapy in non-small-cell lung cancer (NSCLC): Current status and future perspectives. Cancer Immunol. Immunother..

[61] Carlisle J.W., Wolner Z., Pannu S., Mitchell C., Hsu M., Aijaz A., Johnson M., Naqash A.R. (2025). Bispecific Antibodies in Non-Small Cell Lung Cancer: From Targeted Innovation to Real-World Integration. Am. Soc. Clin. Oncol. Educ. Book.

[62] Frentzas S., Austria Mislang A.R., Lemech C., Nagrial A., Underhill C., Wang W., Wang Z.M., Li B., Xia Y., Coward J.I.G. (2024). Phase 1a dose escalation study of ivonescimab (AK112/SMT112), an anti-PD-1/VEGF—A bispecific antibody, in patients with advanced solid tumors. J. Immunother. Cancer.

[63] Wang F., Wei X., Zheng Y., Wang J., Ying J., Chen X., Luo S., Luo H., Yu X., Chen B. (2025). Safety, Pharmacokinetics, and Pharmacodynamics Evaluation of Ivonescimab, a Novel Bispecific Antibody Targeting PD-1 and VEGF, in Chinese Patients with Advanced Solid Tumors. Cancer Med..

[64] Qian Z., Wang G.Y., Henning M., Chen Y. (2024). Measurements of traditional Chinese medicine health literacy regarding chronic pain: A scoping review. BMC Complement. Med. Ther..

[65] Li S.H., Li L., Yang R.N., Liang S.D. (2020). Compounds of traditional Chinese medicine and neuropathic pain. Chin. J. Nat. Med..

[66] Wei Z., Chen J., Zuo F., Guo J., Sun X., Liu D., Liu C. (2023). Traditional Chinese Medicine has great potential as candidate drugs for lung cancer: A review. J. Ethnopharmacol..

[67] Jiang Y., Liu L.S., Shen L.P., Han Z.F., Jian H., Liu J.X., Xu L., Li H.G., Tian J.H., Mao Z.J. (2016). Traditional Chinese Medicine treatment as maintenance therapy in advanced non-small-cell lung cancer: A randomized controlled trial. Complement Ther. Med..

[68] Li Z., Feiyue Z., Gaofeng L. (2021). Traditional Chinese medicine and lung cancer—From theory to practice. Biomed Pharmacother.

[69] Chen H., Zheng M., Zhang W., Long Y., Xu Y., Yuan M. (2022). Research Status of Mouse Models for Non-Small-Cell Lung Cancer (NSCLC) and Antitumor Therapy of Traditional Chinese Medicine (TCM) in Mouse Models. Evid. Based Complement. Altern. Med..

[70] Meyer M.L., Fitzgerald B.G., Paz-Ares L., Cappuzzo F., Jänne P.A., Peters S., Hirsch F.R. (2024). New promises and challenges in the treatment of advanced non-small-cell lung cancer. Lancet.

[71] Ranjan T., Podder V., Margolin K., Velcheti V., Maharaj A., Ahluwalia M.S. (2024). Immune Checkpoint Inhibitors in the Management of Brain Metastases from Non-Small Cell Lung Cancer: A Comprehensive Review of Current Trials, Guidelines and Future Directions. Cancers.

[72] Hayashi H., Chamoto K., Hatae R., Kurosaki T., Togashi Y., Fukuoka K., Goto M., Chiba Y., Tomida S., Ota T. (2024). Soluble immune checkpoint factors reflect exhaustion of antitumor immunity and response to PD-1 blockade. J. Clin. Investig..

[73] Bessede A., Peyraud F., Besse B., Cousin S., Cabart M., Chomy F., Rey C., Lara O., Odin O., Nafia I. (2024). TROP2 Is Associated with Primary Resistance to Immune Checkpoint Inhibition in Patients with Advanced Non-Small Cell Lung Cancer. Clin. Cancer Res..

[74] Freitas-Dias C., Gonçalves F., Martins F., Lemos I., Gonçalves L.G., Serpa J. (2024). Interaction between NSCLC Cells, CD8^+^ T-Cells and Immune Checkpoint Inhibitors Potentiates Coagulation and Promotes Metabolic Remodeling-New Cues on CAT-VTE. Cells.

[75] Benariba M.A., Hannachi K., Zhu S., Zhang Y., Wang X., Zhou N. (2024). A liposome-based assay for cancer biomarker detection: Exploring the correlation between platelet-derived microvesicles and NSCLC-associated miRNAs. Nanoscale.

